# Improvement and transcriptome analysis of root architecture by overexpression of *Fraxinus pennsylvanica DREB2A* transcription factor in *Robinia pseudoacacia* L. ‘Idaho’

**DOI:** 10.1111/pbi.12509

**Published:** 2016-01-25

**Authors:** Yu Xiu, Arshad Iqbal, Chen Zhu, Guodong Wu, Yanping Chang, Na Li, Yu Cao, Wenbiao Zhang, Huiming Zeng, Shouyi Chen, Huafang Wang

**Affiliations:** ^1^ College of Biological Sciences and BiotechnologyNational Engineering Laboratory for Tree BreedingBeijing Forestry UniversityBeijingChina; ^2^ Yanqing No. 1 Vocational SchoolBeijingChina; ^3^ Institute of Genetics and Developmental BiologyChinese Academy of SciencesBeijingChina

**Keywords:** *DREB2A*, *Robinia pseudoacacia*, root architecture, yeast one‐hybrid, vertical root, transcriptome analysis

## Abstract

Transcription factors play a key role to enable plants to cope with abiotic stresses. DREB2 regulates the expression of several stress‐inducible genes and constitutes major hubs in the water stress signalling webs. We cloned and characterized a novel gene encoding the FpDREB2A transcription factor from *Fraxinus pennsylvanica*, and a yeast activity assay confirmed its DRE binding and transcription activation. Overexpression of *FpDREB2A* in *R*. *pseudoacacia* showed enhanced resistance to drought stress. The transgenic plant survival rate was significantly higher than that of WT in soil drying and re‐watering treatments. Transgenic lines showed a dramatic change in root architecture, and horizontal and vertical roots were found in transgenic plants compared to WT. The vertical roots penetrated in the field soil to more than 60 cm deep, while horizontal roots expanded within the top 20–30 cm of the soil. A physiological test demonstrated that chlorophyll contents were more gradually reduced and that soluble sugars and proline levels elevated more sharply but malondialdehyde level stayed the same (*P *<* *0.05). Plant hormone levels of abscisic acid and IAA were higher than that of WT, while gibberellins and zeatin riboside were found to be lower. The root transcriptomes were sequenced and annotated into 2011 differential expression genes (DEGs). The DEGs were categorized in 149 pathways and were found to be involved in plant hormone signalling, transcription factors, stimulus responses, phenylalanine, carbohydrate and other metabolic pathways. The modified pathways in plant hormone signalling are thought to be the main cause of greater horizontal and vertical root development, in particular.

## Introduction

Black locust (*Robinia pseudoacacia* L.), a native tree of North America (Zhang *et al*., 2007), characterized as a nitrogen‐fixing leguminous forest tree species with fast growth, rapid propagation, hard texture and the ability to adapt to different sites and climates (Li *et al*., 2013a). It was introduced to China in 1877–1878, 1897 and the late 1990s (Pan and You, 1994), and adopted as an important reforestation tree species, it is now found in 27 provinces and autonomous regions (Zhang *et al*., 1990). The tree is planted in arid areas of China, such as Loess plateau Shaanxi, Gansu, Qinghai Provinces, Ningxia Hui Autonomous Region and others where the plant showed partial abiotic stress tolerance. Water‐deficit condition changed the morphology and lifespan of the species, that is the plant becomes dwarfed, and age earlier than expected.

Black locust show partial tolerance to cope abiotic stress especially to water‐deficit condition. But due to the tree morphology such as thin pinnate leaves, water loss by transpiration is high in severe drought stress condition (Yang *et al*., 2006). Keeping this water‐deficit condition in mind, the rooting mechanism is an important factor to also be studied. The tree is shallow rooted, and most of its roots are found in the top 20–30 cm soil layer (Zhang and Xu, 2011). Plants have the potential to enhance abiotic stress tolerance in over and underground organs. Roots have long been proposed as a best choice of research to improve crop adaptation to water stress condition (Vadez, 2014). The simple supposition is that more profuse root systems could absorb more moisture from surrounding soil, while vertical‐extensive roots absorb water more efficiently from moist soil found at deeper levels, in particular (Grieder *et al*., 2014). Various factors such as genetic (Kell, 2011) and hormonal control (Santner *et al*., 2009) influence root growth and root system expansion. It is crucial to improve drought resistance in *R*. *pseudoacacia* for reclaiming up arid lands by utilizing genetic engineering techniques.

Molecular breeding has been performed by manipulating the genes to improve the structure and functions of cellular components (Wang *et al*., 2003). A multidisciplinary technique with thriving manipulation of a whole range of omics technologies will play an important role in drought tolerance (Tripathi *et al*., 2014). *Cis*‐acting elements of the promoter region interact with transcription factors (TFs) of the gene alter the whole cascade of genes to enhancing abiotic tolerance (Akhtar *et al*., 2012). The main TFs in water stress signalling network include DREB, bHLH, MYB, ERF, bZIP and WRKY TFs (Docimo *et al*., 2013; Jiang and Deyholos, 2009; Tripathi *et al*., 2014). The limited similar genes have been identified from woody plants.

The *DREB* (dehydration‐responsive element‐binding proteins) gene family mainly plays key role in plant stress response mechanisms (Lata *et al*., 2011). *DREB1* (Stockinger *et al*., 1997) is related to cold‐resistance, while *DREB2* gene family is particularly important in responses to drought, salinity and heat stress (Liu *et al*., 1998; Xu *et al*., 2011). *DREB* gene family plays a significant job in the ABA‐independent stress signal response mechanisms that stimulate the expression of multiple abiotic stress‐responsive genes in plants. Initial data about cDNAs encoding DRE‐binding proteins, CBF1 (CRT‐binding factor1), DREB1A and DREB2A were isolated from *Arabidopsis* (Liu *et al*., 1998). Following on from this, several *DREB* genes have been reported from various plants (Sadhukhan *et al*., 2014; Yang *et al*., 2011). These TFs particularly bind to the DRE sequence and activate the expression of several down‐regulated genes driven by it.

*Agrobacterium*‐mediated transformation is the most utilized method used in genetic engineering (Rai *et al*., 2012). This technique utilizes the natural ability of bacterium for integrating specified DNA fragments into the host genome, additionally the transgenic plants tag along the Mendelian inheritance (Subramanyam *et al*., 2013). Appropriate marker for the selection of transgenic lines is essential for plant (Yau and Stewart, 2013), various reports showed *GUS* as a selection marker for *Agrobacterium*‐mediated transformation into black locust (Han *et al*., 2000; Igasaki *et al*., 2000; Kanwar *et al*., 2003). Choice of suitable promoter is essential for the successful genetic manipulation and the expression of an exogenous gene to achieve the desire goal (Roy *et al*., 2000). *DREB1A* gene expression driven by structure promoter, *35S* has been reported to improve plant drought resistance, but led to dwarfed phenotype (Kasuga *et al*., 1999). Whereas inducible promoter, such as *rd29A* with the DRE/CRT *cis*‐acting element from *Arabidopsis thaliana,* induces the drought stress gene expression (Yamaguchi‐Shinozaki and Shinozaki, 1994) and improves the soya bean drought resistance and morphological normal growth (de Paiva Rolla *et al*., 2014). A limited amount of similar research has been performed in perennial woody plants.

Biosafety issues are parallel with crop engineering, particularly utilization of selectable markers adversely affect the organisms. Constitutive promoter, such as CaMV *35S,* mostly used in plant gene transformation, but risk is involve in the utilization of such promoter as have chance to release the transgene into the environment through horizontal gene flow (Shah *et al*., 2015). The infertile transgenic acceptors are important for reducing biosafety risk in black locust transformation. Generally, the black locust is reproduced by seeds, which may lead horizontal gene flow into nontarget organisms and soil (Talas‐Oğras, 2011). Understudy cultivar, *R. pseudoacacia* ‘Idaho’ with spectacular purple raceme blooms as black locust (Guo *et al*., 2006; Li *et al*., 2006) but infertile, with no seed production (data not shown). The cultivar offspring produces easily with root suckers. The infertile transgenic receptor of black locust reduces the risks of GMO biosafety by gene flow via gamogenesis.

Drought stress as a vital environmental factor, sever and prolong existence of drought stress could retard plant growth and development, this situation is alarming for plant biotechnologists. This study describes the transformation of *FpDREB2A* to *R. pseudoacacia* ‘Idaho’ with comprehensive physiological and transcriptome analysis for enhancing drought stress through improved roots. Root architecture and endogenous plant hormone (ABA, IAA, GA and ZR) were examined and compared with WT plants. Differentially expressed genes (DEGs) of transgenic plant roots and WTs were analysed using high‐throughput sequencing and confirmed by RT‐qPCR. Furthermore, the DEGs regulation bio‐pathways of vertical root architecture were studied utilizing Gene Ontology (GO), Cluster of Orthologous Groups of proteins (COG), and Kyoto Encyclopedia of Genes and Genomes (KEGG) databases.

## Results

### DRE‐binding and transcriptional activation activities of the DREBs

**Figure 1:**
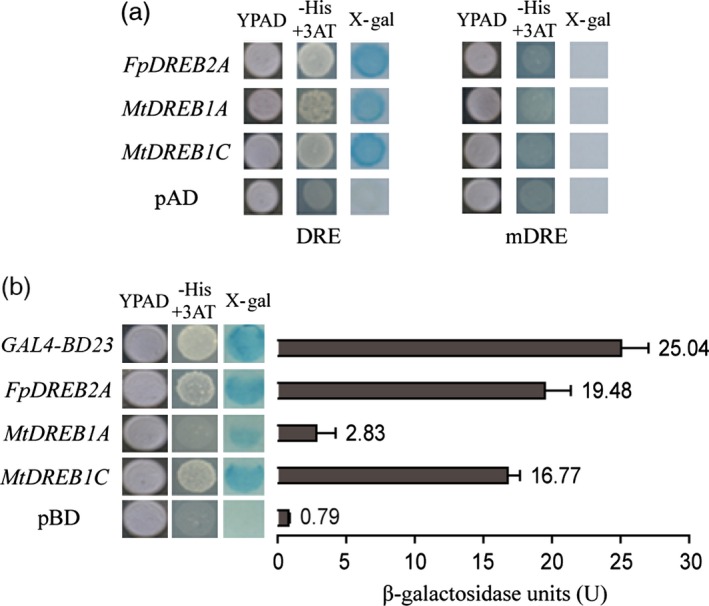
DRE‐binding properties and transcriptional activities of DREBs. (a) Growth of DRE and mDRE carrying plasmid pAD, pAD‐*FpDREB2A*, pAD‐*MtDREB1A* and pAD‐*MtDREB1C* on YPAD and selective YPAD culture medium, and the result of colony‐lift filter assay. (b) Growth of YRG‐2 transformed with plasmid pBD, pBD‐*GAL4‐BD23*, pBD‐*FpDREB2A*, pBD‐*MtDREB1A* and pBD‐*MtDREB1C* on YPAD and selective YPAD culture medium, and the determination of β‐galactosidase reaction (*n* = 3). Individual values as an average of three replicates and error bar represent standard deviations (SD).

The two yeast strains with *cis*‐acting elements DRE (TACCGACAT) and mDRE (TATTTTCAT) were transformed with pAD, pAD‐*FpDREB2A*, pAD‐*MtDREB1A* and pAD‐*MtDREB1C* plasmids, inoculated on selective YPAD culture medium (Figure 1a). Yeast DRE carrying pAD‐*DREB* plasmids grew on the selective YPAD medium and showed blue colonies on colony‐life filter paper, that is they have *lac Z* activity. In contrast, mutant yeast mDRE failed to grow on the selective medium. Results showed that the candidate target genes encoded FpDREB2A, MtDREB1A and MtDREB1C could specifically bind to DRE element containing – TACCGACAT – core sequence *in vitro*.

To detect the transactivation ability of DREBs, the coding regions of the genes were cloned into the YRG‐2 yeast expression vector pBD and tested by culturing on selective media (Figure 1b). Yeast YRG‐2 carrying the pBD‐*GAL4‐BD23* (positive control), pBD‐*FpDREB2A*, pBD‐*MtDREB1A* and the pBD‐*MtDREB1C* plasmids showed growth on YPAD medium. β‐galactosidase activities of the four strains were quantified as 25.04 U, 19.48 U, 2.83 U and 16.77 U, respectively. While YRG‐2/pBD could not grow on the selective medium with β‐galactosidase activity 0.79 U. FpDREB2A showed the highest activity followed by GAL4‐BD23.

### The transformation and screening of transgenic *R. pseudoacacia* ‘Idaho’

**Figure 2:**
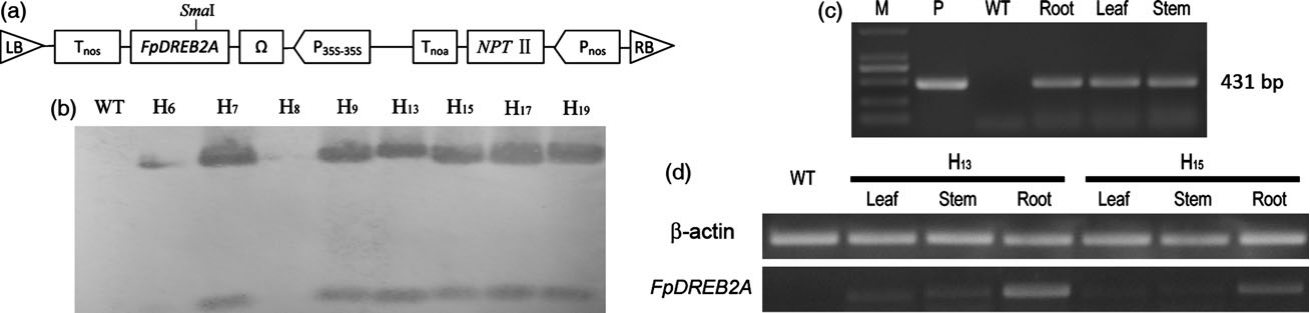
The transformation and transgene stability of *FpDREB2A* in transgenic *R. pseudoacacia* ‘Idaho’. (a) T‐DNA region of binary vector pBin438. (b) Southern blotting analysis of *FpDREB2A* in transgenic *R. pseudoacacia* ‘Idaho’: WT (lane 1) and transgenic plant (lane 2–9), in which lane 2 and 4 showed negative hybridization, while lane 3 and 5–9 showed positive hybridization. (c) Determination of *FpDREB2A* in roots, stems and leaves of 5‐year‐old transgenic plants by PCR analysis. (d) Relative transcript levels of *FpDREB2A* in different tissues of transgenic plants.

T‐DNA including *FpDREB2A* under the expression of *P*_*35S‐35S*_ promotor shown as Figure 2a was delivered in *R*. *pseudoacacia* ‘Idaho’ through *Agrobacterium*‐mediated transformation system. Successful integration of the transgene was assessed by PCR (data not shown) and confirmed by Southern blot analysis, six of eight transgenic lines showed positive hybridization on agarose gel electrophoresis (Figure 2b). After 5 years growing in field (Figure S1) under natural condition (−33 °C and 441.9 mm annual precipitation, data recorded by the field weather station), plants were subjected to test *FpDREB2A* in the genomic DNA of the roots, leave and stems (Figure 2c), and its transcription was confirmed by RT‐PCR (Figure 2d).

### Drought resistance of the transgenic *R. pseudoacacia* ‘Idaho’

**Figure 3:**
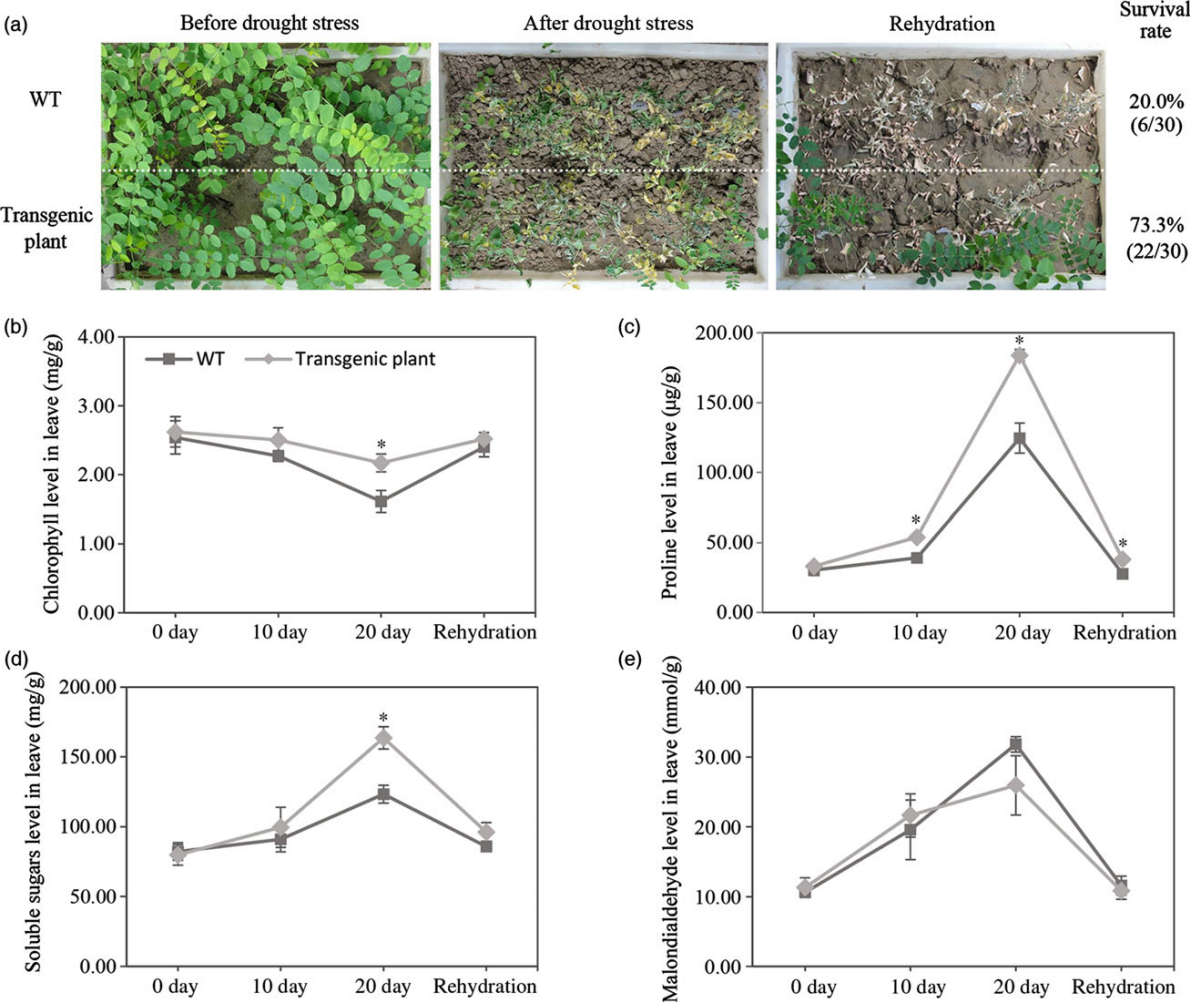
Drought stress tolerant of and physiological index of transgenic *R. pseudoacacia* ‘Idaho’. (a) Drought stress tolerant of WT and transgenic plant. WT and transgenic root suckers of same age and size were transplanted to a box and watered for revival; thereafter, water was withheld for 28 days; then plants were re‐watered for 21 days. Recovery rates were calculated from three independent experiments (*n* = 10 for each experiment). (b) Drought‐induced chlorophyll, proline, soluble sugars and MDA levels in leaves of WT and transgenic lines after drought treatment (0, 10 and 20 days) and after re‐water treatment (20 days). Individual values as an average of three replicates and error bar represent standard deviations (SD). Asterisks (*) indicated statistically significant difference between WT and transgenic plants, as determined by Student's *t*‐test (*P *<* *0.05).

Survival rate of transgenic plants and WT was recorded approximately 73.3% and 20.0%, respectively (Figure 3a) under soil drying and re‐watering treatments. There was a fourfold increase in the number of plants that survived relative to WT for transgenic plants. Based on drought resistance test, two transgenic lines H_13_ and H_15_ were selected for further analysis.

Some physiological aspects of the transgenic plants in response to drought stress were compared with the WT (Figure 3b–e). The chlorophyll content decreased more gradually, while the contents of proline and soluble sugars elevated more sharply in leaves but malondialdehyde level stayed the same with compared to WT (*P *<* *0.05). Result showed the same level of the mentioned four indexes in the leaves of transgenic and WT plants in response to re‐watering.

### Root architecture of transgenic *R. pseudoacacia* ‘Idaho’ in the field

**Figure 4:**
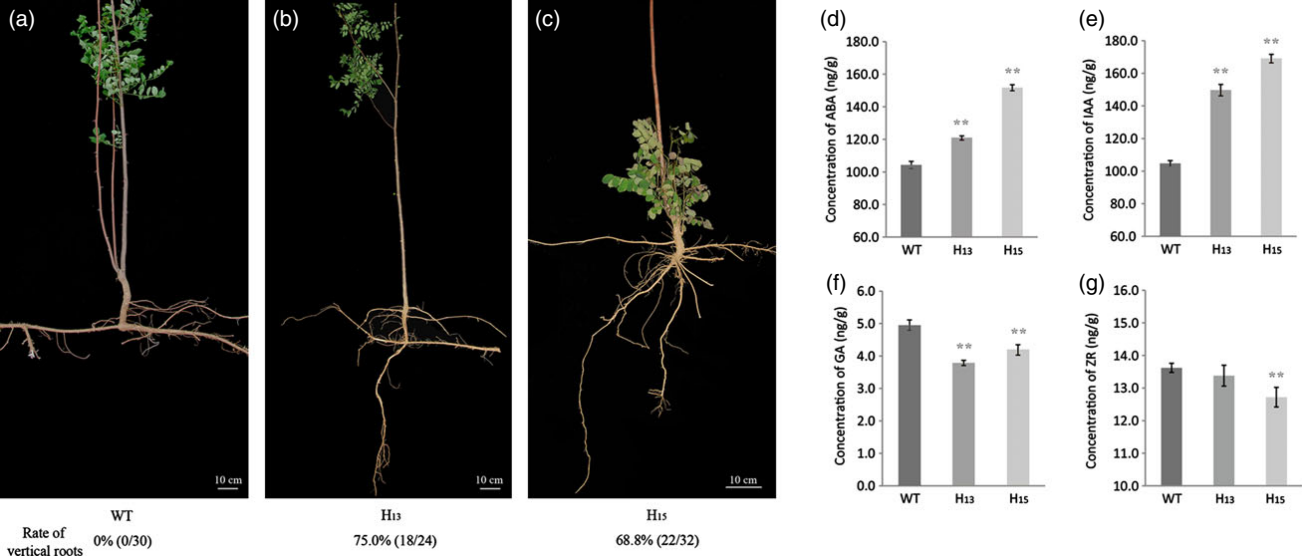
Root morphology and plant hormone levels in roots of transgenic *R. pseudoacacia* ‘Idaho’ of about 5 years old. (a) Horizontal roots of WT (*n* = 30). (b and c) Horizontal and mainly vertical roots of transgenic lines H_13_ (*n* = 24) and H_15_ (*n* = 32), respectively. Scale bars: (a–c), 10 cm. Levels of (d) ABA, (e) IAA, (f) GA and (g) zeatin riboside (ZR) in roots (concentration of plant hormones based on fresh weight). The results are shown for triplicate experiments, and error bars represent SD. Double asterisks indicated statistically significant difference between groups, as determined by Student's *t*‐test (*P *<* *0.01).

The root morphology of *FpDREB2A* transgenic *R. pseudoacacia* ‘Idaho’ changed dramatically (Figure 4). The diameters of root–stem transition zones at soil surface were about 2 cm for both of the transgenic and the WT *R. pseudoacacia* ‘Idaho’. The WT (Figure 4a) and vector control (figure not shown) plant roots were horizontally distributed and concentrated mainly in the top 20–30 cm of the field soil. But the transgenic plants developed both horizontal and vertical roots, which extended more than 60 cm into the soil (Figure 4b,c).

### Plant hormones analysis of root architecture change in *R. pseudoacacia* ‘Idaho’

Plant hormones ABA, IAA, GA and ZR were studied in the root development regulation of transgenic plant as shown in Figure 4. ABA contents (Figure 4d) in the transgenic plant roots were significantly higher than that of WT plants (*P *<* *0.01), transgenic line H_15_ ABA content was 151.71 ng/g, which was 1.45 times of that in WT plants. While GA levels (Figure 4e) were significantly lower than that of WT plants (*P *<* *0.01). H_13_ had the lowest (3.79 ng/g) GA content, which was 76.6% of WT plants.

IAA level of transgenic plant was recorded more than that of WT plants as shown in Figure 4f. IAA levels in line H_13_ and H_15_ roots were 1.42 times and 1.61 times of WT (*P *<* *0.01), respectively. H_15_ had the highest of IAA level (169.02 ng/g).

In contrast to ABA and IAA, plant hormone ZR contents in the roots of transgenic lines were found to be lower than that of WT plants. ZR contents of transgenic line H_15_ was found significantly different (*P *<* *0.01) than that of WT.

### Transcriptome sequencing and *de novo* assembly of transgenic *R. pseudoacacia* ‘Idaho’

We used the Illumina HiSeq^™^ 2000 high‐throughput sequencing platform to sequence transcriptomes obtained from vertical roots of transgenic *R. pseudoacacia* ‘Idaho’ and horizontal roots of WT plant. A total of 12 682 813 and 11 055 829 clean reads (2.52 and 1.99 Gb nucleotides) were obtained from WT (T_1_) and transgenic (T_2_) cDNA libraries, respectively. Guanine–cytosine (GC) contents were recorded 43.8% and 43.2%, and both Q20 percentages were higher than 95% (Table 1).

**Table 1:** Statistics of sequencing data

Sample	Total reads	Total nucleotides (nt)	Q20 percentage	GC percentage
T_1_	12 682 813	2 522 190 853	95.30%	43.80%
T_2_	11 055 829	1 992 032 645	96.49%	43.22%

Summary statistics for the sequencing of T_1_ and T_2_ cDNA libraries. Q20 percentage denotes the percentage of base error rate below 0.01.

The raw reads were processed by removing low quality reads, adapter sequences and possible contaminated reads to generate high quality of clean reads (Figure S2). For assembling the clean reads, we used SOAP‐de novo software (Li *et al*., 2009) and Trinity software (Grabherr *et al*., 2011), 70 814 and 69 089 transcripts were obtained from T_1_ and T_2_ cDNA libraries. All of the assembled transcripts were longer than 200 bp (Table 2) with N50 length of 1206 and 1094 bp, mean length of 785 and 751 bp, and total length of 55.63 and 51.91 Mb. Finally, the two cDNA libraries generated 56 195 unigenes with N50 of the assembled sequence was 1004 bp, with mean length of 653 bp and a total length of 36.71 Mb. The long unigenes (>1 kb) identified accounted for 19.2% of the total unigenes.

**Table 2:** Assembling result of *R. pseudoacacia* ‘Idaho’ transcriptome

	T_1_ transcripts	T_2_ transcripts	All unigenes
200–300 nt	18 297 (25.84%)	16 378 (23.71%)	18 916 (33.66%)
300–500 nt	16 748 (23.65%)	16 775 (24.28%)	15 438 (27.47%)
500–1000 nt	16 875 (23.83%)	18 279 (26.46%)	11 055 (19.67%)
1000–2000 nt	14 130 (19.95%)	14 318 (20.72%)	8161 (14.52%)
≥2000 nt	4764 (6.73%)	3339 (4.83%)	2625 (4.67%)
N50 length	1206	1094	1004
Mean length	785.55	751.41	653.21
Total number	70 814	69 089	56 195
Total length	55 628 066	51 914 254	36 707 029

### Structural information and functional annotation of the unigenes

To obtain structural information, Getorf software (Mortazavi *et al*., 2008) was used for predicting ORF of the total unigenes and their coding sequences and protein sequences. Unigenes with more than 1 kb length were selected to analysis simple sequence repeats (SSRs) by MISA software (Kanehisa *et al*., 2008).

A total of 55 943 protein‐coding genes were predicted in the transcriptome library. Approximately, 52% of amino acid sequences related to open reading frames (ORFs) was longer than 200 aa, while the remainder were less than 200 aa (Table S2). SSRs analysis results showed the occurrence of total 4361 SSRs of 10 786 unigenes examined in *R. pseudoacacia* L. ‘Idaho’ transcriptome as shown in Table S3. The mono‐nucleotide SSRs was the most prevalent group of markers (51.4%) followed by tri‐nucleotide (24.6%) and di‐nucleotide (22.8%) SSRs. Only a small fraction of tetra‐, penta‐ and hexa‐nucleotide SSRs were found in the assembled unigenes.

The transcriptional information was obtained by BLAST software, and the unigenes were assigned to NR, NT, Swiss‐Prot, TrEMBL, GO, COG and KEGG database (Table 3). A total of 42 685 unigenes were identified, in which 26 908 unigenes were enriched in GO database (Figure S3), 9546 unigenes were analysed by COG (Figure S4), and 7462 unigenes were mapped in KEGG pathways (Figure S5).

**Table 3:** Summary of unigene annotations

Annotated databases	All sequence	>300 bp	>1000 bp
Total	42 685	21 363	10 724
NR	36 516 (85.55%)	18 434	10 634
NT	39 994 (93.70%)	19 968	10 636
Swiss‐Prot	24 857 (58.23%)	11 623	9084
TrEMBL	37 258 (87.29%)	18 799	10 652
GO	26 908 (63.04%)	13 117	8974
COG	9546 (22.36%)	3985	4540
KEGG	7462 (17.48%)	3398	2835

### Gene ontology network analysis of *R. pseudoacacia* ‘Idaho’ roots

The unigene expression levels of the two transgenic lines were analysed using IDEG6 software (Romualdi *et al*., 2003) and normalized by the number of fragments per kilo base exon region per million mapped reads (FPKM), (Trapnell *et al*., 2010), following this method 2011 differential expression genes (DEGs) of 56 195 unigenes were annotated. Compared with T_1_, 995 unigenes were up‐regulated and 1016 were down‐regulated in T_2_ library (Figure S6).

**Figure 5:**
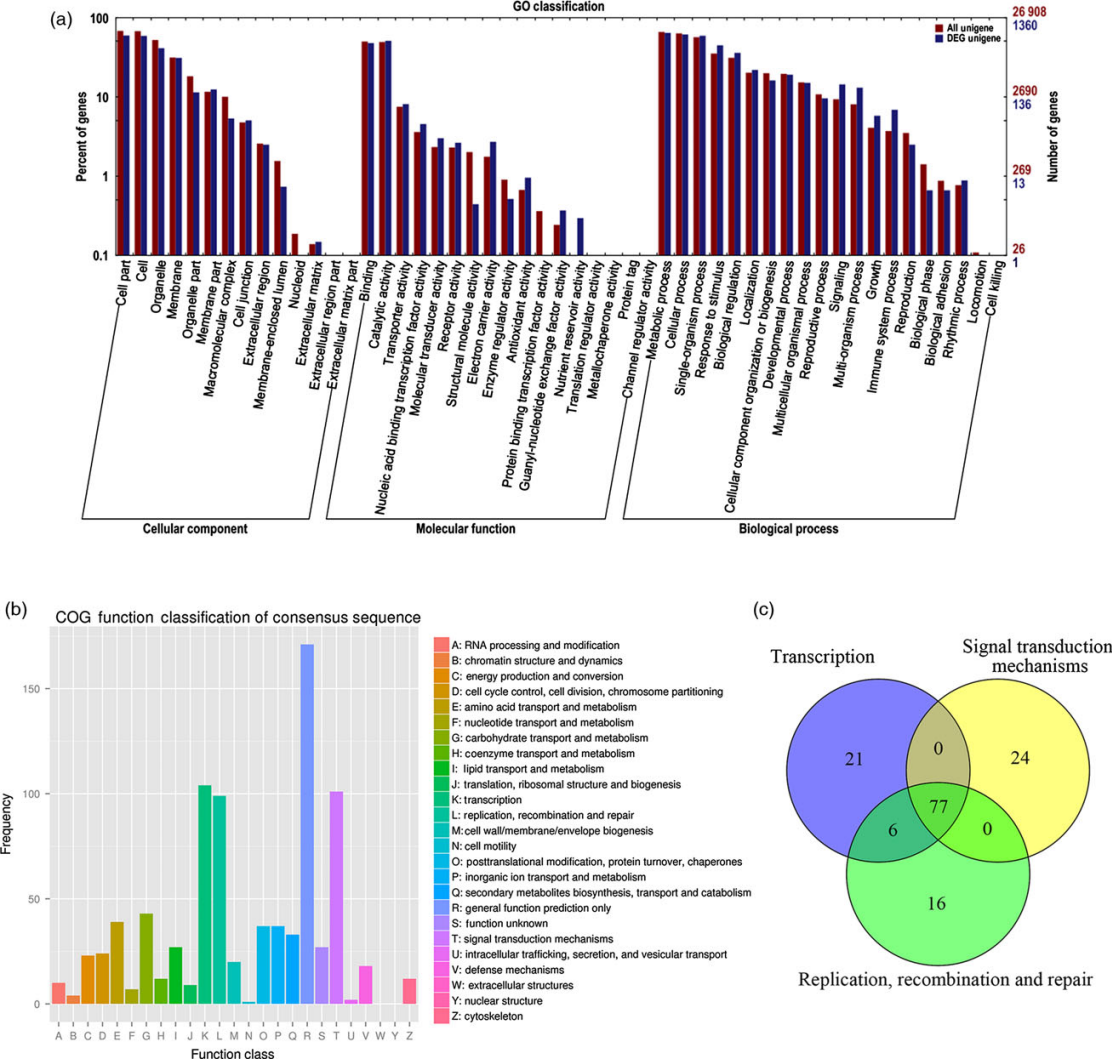
Analysis of transcriptome in *R. pseudoacacia* ‘Idaho’ roots. (a) Functional classifications of GO terms of all DEGs. FPKM method was used to calculate the levels of gene expression. Of 2011 DEGs, functional classifications of GO terms of 1360 DEGs were classified into three categories. (b) COG classification of DEGs. A total of 500 DEGs were annotated into 23 groups based on COG database, and 860 annotations were obtained. (c) Venn diagram of the number of DEGs classified into groups of ‘transcription’, ‘signal transduction mechanisms’, and ‘replication, recombination and repair’.

The functions of the DEGs were searched by GO database for plotting annotation results. Of 2011 DEGs, a total of 1360 DEGs were categorized into three main categories (cellular component, molecular function and biological process) and 51 functional groups (See Figure 5a). Compared with T_1_, 623 unigenes were up‐regulated and 737 down‐regulated in T_2_ library. The percentage of DEGs in ‘response to stimulus’, ‘biological regulation’ and ‘signalling’ groups were higher than that of all unigenes in the three groups, showing a more important role in growth regulation of roots. Annotations of DEGs against the GO database showed that the FpDREB2A enhanced the expression of many genes related to stress response, such as ABA response element‐binding protein (AREB) and heat‐shock protein (Table S4).

### COG enrichment analysis of DEGs in *R. pseudoacacia* ‘Idaho’ roots

Furthermore, the DEGs were annotated based on COG analysis. 500 DEGs, less than GO results, had 860 annotations and were classified into 23 groups (Figure 5b). Among these groups, approximately 20% of DEGs cannot be annotated accurately and therefore classified as the cluster of ‘general function prediction only’. The number of genes involved in ‘transcription’, ‘signal transduction mechanisms’ and ‘replication, recombination and repair’ were about 12%, which were the largest according to COG database. Interestingly, 77 unigenes were found to be classified into the three clusters simultaneously (Figure 5c) and many regulated DEGs had serine/threonine‐protein kinase or mitogen‐activated protein kinase activities, which also response to stress and plant hormone stimulus (Table S5).

### Comparative transcriptome analysis of root architecture by KEGG pathways analysis

**Figure 6:**
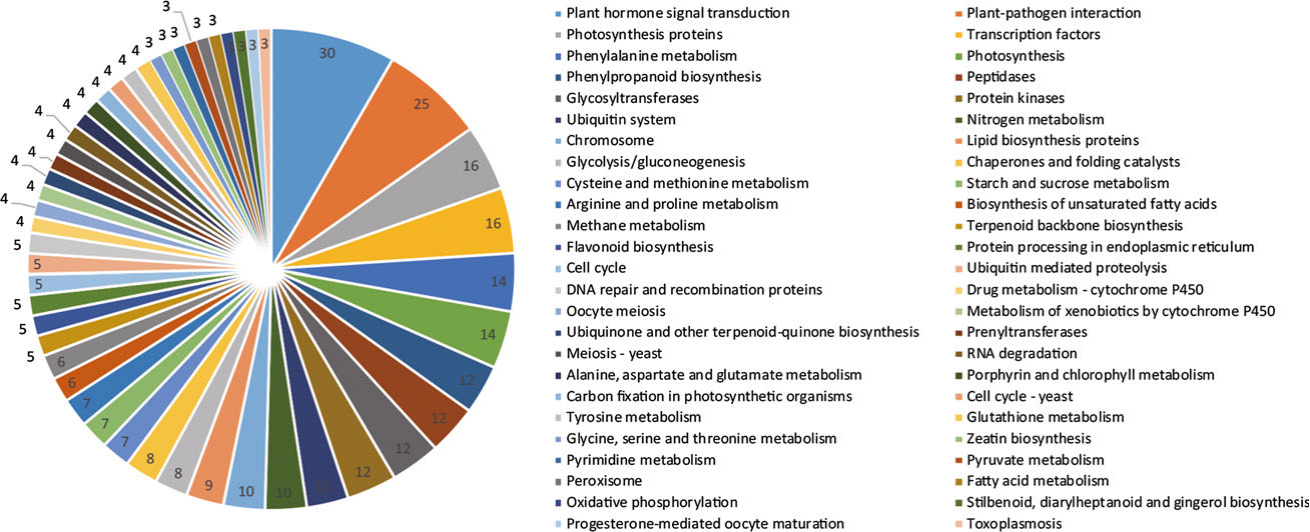
Functional characterization of transgenic *R. pseudoacacia* ‘Idaho’ roots for KEGG pathways. The DEGs were classified in top 50 KEGG pathways, and area under each pie shows the value in number.

The DEGs regulation pathways were analysed by KEGG for the gene functions and genomic information (Kanehisa and Goto, 2000) and 260 DEGs (including 516 unigenes) were found to be involved into 149 KEGG pathways (Table S6). The top 50 enriched pathways ranked in KEGG pathways were independently showed in Figure 6. Most of the DEGs were enriched in plant hormone signal transduction, plant–pathogen interaction, transcription factor pathways, protein kinases and metabolism of energy substances.

**Figure 7:**
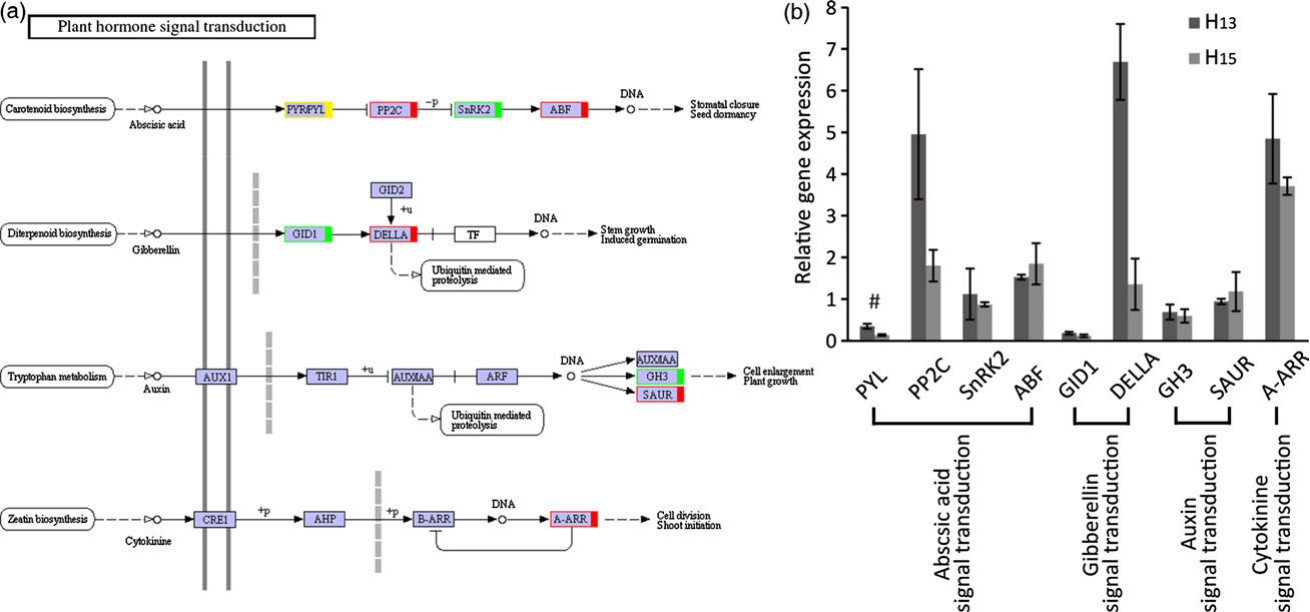
Analysis of DEGs related to plant hormones in transgenic plant roots. (a) KEGG analysis of abscisic acid, gibberellin, auxin and cytokinine signal transduction. Red/green indicated that the DEGs were up‐/down‐regulation, respectively. Yellow showed that the expression level was different in transcriptome and RT‐qPCR analysis. (b) Relative expression analysis of DEGs by RT‐qPCR. The data showed the relative ratios of gene expression in transgenic plant roots compared with WT. Eight of the 9 DEGs (89%) were verified to be changed by RT‐qPCR analysis. The results are shown for triplicate experiments and error bar represent SD.

**Figure 8:**
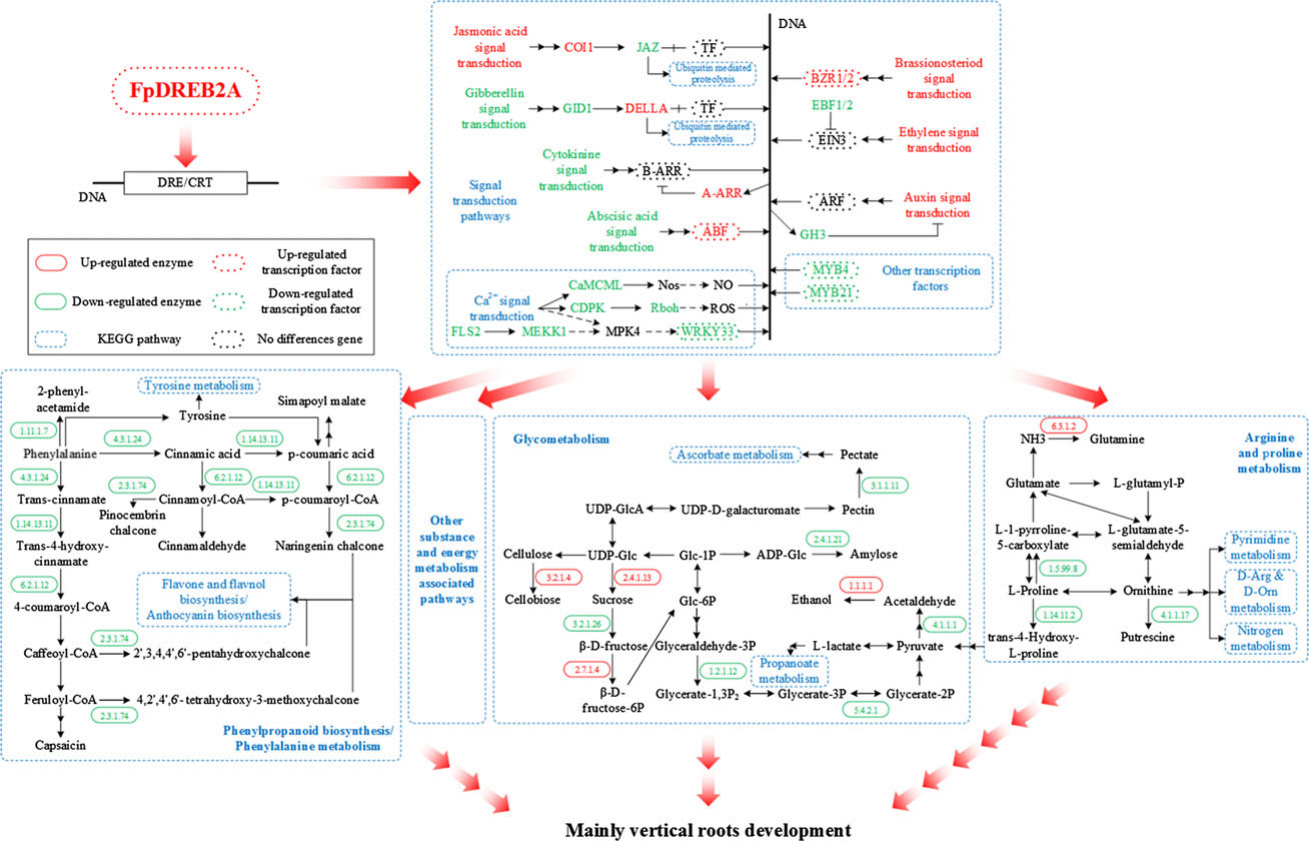
A diagram of the main pathways regulated by FpDREB2A according to KEGG enrichment. Eleven modified pathways (plant hormone signal transduction, plant–pathogen interaction, transcription factors, protein kinases, glycolysis/gluconeogenesis, phenylalanine metabolism, phenylpropanoid biosynthesis, starch and sucrose metabolism, arginine and proline metabolism, flavonoid biosynthesis, stilbenoid, diarylheptanoid and gingerol biosynthesis) were assembled into this diagram.

Plant hormone regulation pathways ranked first in 149 KEGG pathways, including abscisic acid, gibberellin, auxin, cytokinin (Figure 7a and Table S7), ethylene, brassinolide and jasmonic acid pathways (Figure 8) . To validate the transcriptional pattern identified by KEGG pathway, expression of 9 differentially expressed genes was analysed using RT‐qPCR. The results showed that the tested genes (8 of 9 genes) followed the expression pattern observed in RNA sequencing (Figure 7b) except for *PYR/PYL*, validating the KEGG pathways.

The DEGs regulation pathways analysed for ABA by KEGG showed the involvement of four DEGs, that is *PYR*/*PYL*,* PP2C*,* SnRK2* and *ABF*, representing the main core components to complete the ABA regulation of gene (Fujii *et al*., 2009). Results showed that ABA signal pathways were changed in transgenic *R. pseudoacacia* ‘Idaho’ but the mechanisms involved are still unclear. Mentioned DEGs in our work were verified by RT‐qPCR and results showed that *PP2C* and *ABF* were up‐regulated while *PYL* was down‐regulated. The core components for ABA signalling, PYL‐ PP2C‐ SnRK2‐ABF function as ABA receptors, repressors, positive regulators, and ABRE‐binding factors, expressed in an interval of down‐ and up‐regulation (Figure 7a). This suggested that the transgenic roots regulated ABA signal transduction and supposed to be utilize in the root architecture.

GA‐GID1‐DELLA complexes acted as a pivotal regulator in GA signalling. In GA signalling pathway, *GIDI* was found to down‐regulated, meanwhile, *DELLA* up‐regulated (Figure 7a). This suppressed GA binding to receptor GIDI and diminished GA responses by DELLA which function as repressors in GA signalling pathways. DEGs in auxin pathway (Figure 7a) included Gretchenhagen‐3 (*GH3*) and Small auxin‐up RNA (*SAUR*). Our results revealed that *GH3* expression was down‐regulated and *SAUR* was slightly up‐regulated (RT‐qPCR analysis). A‐ARR was up‐regulated in cytokinin pathway (Figure 7a), and result showed that ZR concentration significantly decreased in transgenic lines (Figure 4g).

Furthermore, five DEGs were enriched into three pathways, in which *EBF1/2* was down‐regulated in ethylene pathway, *BRI1* and *BZR1/2* were up‐regulated in brassinosteroid pathway and up‐regulated *COI1* and down‐regulated *JAZ* were found in jasmonic acid signalling. While no DEG enriched in salicylic acid pathway.

Following hormone regulation pathways, phenylropanoid biosynthesis/phenylalanine metabolism, glycometabolism, arginine and proline metabolism were found the main pathways regulated by FpDREB2A according to KEGG enrichment. As shown in Figure 8, prolyl 4‐hydroxylase, proline dehydrogenase, ornithine decarboxylase and β‐fructofuranosidase were down‐regulated, while sucrose synthase and endo‐1,4‐β‐d‐glucanase was up‐regulated in transgenic plant root. The results showed that the degradation of proline and sucrose were down‐regulated while the biosynthesis of sucrose and cellobiose was up‐regulated in arginine and proline metabolism and sucrose and starch metabolism.

Phenylalanine biosynthesis and metabolism pathway (e.g. phenylalanine ammonia‐lyase, trans‐cinnamate 4‐monooxygenase and 4‐coumarate‐CoA ligase) was inhibited by down‐regulation of MYB4 transcription factor (Docimo *et al*., 2013; Yang *et al*., 2001), this might restrain lignin biosynthesis.

Glycolysis (e.g. glyceraldehyde 3‐phosphate dehydrogenase and phosphoglycerate mutase) was down‐regulated in the transgenic plant. Several DEGs classified into calcium‐binding protein, calcium‐dependent protein kinase and WRKY33 transcription factor (Jiang and Deyholos, 2009) were found to be regulated in transgenic plant root by KEGG annotation.

## Discussion

Plants have evolved regulatory mechanisms to adapt to environmental water deficit. TFs play diverse and critical roles during plant stress conditions, regulate expression of stress‐responsive genes by binding specifically to the motif of the promoters to modulate resistance to drought (Zhu, 2002). *AtDREB2A* from *Arabidopsis thaliana* (Yamaguchi‐Shinozaki and Shinozaki, 1994) is an important TF that regulates stress‐responsive gene expression through DRE *cis*‐elements, induced by dehydration stress and may activate other genes involved in drought stress tolerance (Liu *et al*., 1998), and play a vital role in providing tolerance to multiple stresses (Chen *et al*., 2009a,b). *AtDREB2* homologous genes have been isolated from economically important cereal crops such as rice, wheat, barley, maize, pearl millet, and foxtail millet (Agarwal *et al*., 2007; Dubouzet *et al*., 2003; Egawa *et al*., 2006; Lata *et al*., 2011; Qin *et al*., 2008; Xue and Loveridge, 2004). Their transcripts were found to be regulated by alternative splicing in barley, wheat, maize and rice with most of them showing transactivation abilities in yeast or plant cells (Agarwal *et al*., 2007; Dubouzet *et al*., 2003; Egawa *et al*., 2006; Qin *et al*., 2008).

In the present study, *FpDREB2A* (AY536056) were cloned from woody plant, *Fraxinus pennsylvanica,* showed 98% sequence similarity to *AtDREB2A* (data not shown). The DREB proteins have DRE binding and transcriptional activation functions (Figure 1), same results reported for *Arabidopsis thaliana* (Stockinger *et al*., 1997), *Triticum aestivum* (Shen *et al*., 2003a), *Atriplex hortensis* (Shen *et al*., 2003b) and *Malus baccata* (Yang *et al*., 2011). The MtDREB1A activity was found lowest which might be due to presence of Ser‐/Thr‐rich residues, a down‐regulator in the coding region (Chen *et al*., 2009a,b). In order to understand the gene functions in response to drought, *FpDREB2A* was transformed to *R. pseudoacacia ‘Idaho’* (Zeng and Wang, 2006), the transgenic plants were confirmed by Southern blotting and showed significantly high recovery rate to drought resistance test (Figure 3). The results supported previous published work that *AtDREB2A* from *Arabidopsis thaliana* have been functional to improve drought tolerance of transgenic plants (Liu *et al*., 1998; Matsukura *et al*., 2010; Shinozaki and Yamaguchi‐Shinozaki, 2007).

The transgenic *R*. *pseudoacacia* ‘Idaho’ dramatically changed root architecture, that is improved root system was observed having horizontal stretch roots like WT additionally the vertical elongated roots (Figure 4a). The results suggest that the *DREB2A* have the potential of vertical root development. Moreover, the *AtDREB* driven by *35S* led to transgenic plants enhanced stress tolerance but retarded growth in some model plants (Liu *et al*., 1998; Matsukura *et al*., 2010). While the *FpDREB2A* transgenic *R*. *pseudoacacia* ‘Idaho’ after 5 years of planting in the field did not showed growth retardation (data not shown). This might be due to the woody perennial tree nature of *R*. *pseudoacacia* and its maintenance in natural field conditions. Plant root morphology is an important feature for soil water absorption (Hodge *et al*., 2009); especially, the long and deep stretch roots were important adaptation for efficient uptake from deep soil in water‐deficit environments (Li *et al*., 2014). Recent studies have shown that root growth is closely connected with drought tolerance (Pennisi, 2008), root length and its architecture govern the adaptability of plants to various stress conditions, including drought stress. The adaptive advantage of increased root length and more number of root hairs that facilitate access to water under drought stress has been reported earlier (Miyazawa *et al*., 2009). Ballif *et al*. (2011) found that overexpressing transcriptional factors enhanced primary root elongation due to a faster cell division and/or elongation, same result of *FpDREB2A* is presented for transgenic black locust in this article.

Mechanisms involved in the plant drought resistance were elucidated in physiology and transcriptome analysis. Physiological tests suggested that the plant hormone alteration (Figure 4d–g) and the higher chlorophyll, proline and soluble sugars accumulation (Figure 3b–d) could enhance plant drought stress resistance in transgenic plants compared with WT. The similar effects were verified in some model plants (Gupta *et al*., 2014; Sperdouli and Moustakas, 2012; Ziaf *et al*., 2011).

Transcriptome analysis provides a meaningful tool for explaining the gene regulations involved in abiotic stress responses and has been utilized for the analysis of several plant including *Arabidopsis thaliana* (Gan *et al*., 2011), *Zea mays* (Ohtsu *et al*., 2007), *Lycopersicum esculentum* (Moxon *et al*., 2008) and others. *R*. *pseudoacacia* whole‐genome sequences and gene annotations are not available at present, reference‐based transcriptome analysis is also not feasible. Therefore, *de novo* assembly appears to be a good approach to study drought regulated expression changes in this species.

Transcriptome analysis of vertical versus horizontal roots in *R*. *pseudoacacia* ‘Idaho’ identified a large number of high‐quality sequences, their corresponding proteins may have special functions in the root architecture and response to drought stress. Predicted ORF of the total unigenes (Table S2), their coding sequences, protein sequences and transcriptional information was obtained by BLAST software and the unigenes were assigned to NR, NT, Swiss‐Prot, TrEMBL, GO (Figure S3), COG (Figure S4) and KEGG (Figure S5) database. The GO analysis indicates the involvement of 2011 DEGs and its cross‐talk among various pathways, especially the involvement of DEGs groups ‘response to stimulus’, ‘signalling’ and ‘biological regulation’ in vertical root development (Figure 5a). Results showed that *FpDREB2A* have the potential to change the whole omics of the drought responsive organ (roots) by altering its metabolic and cellular pathways. Information from cell sorting and metabolic pathways established a network that regulates lateral root outgrowth upon stimulation by abiotic stimuli (Gifford *et al*., 2008).

Based on the KEGG pathways database for gene functions and genomic information (Kanehisa and Goto, 2000), DEGs were enriched into 149 different pathways (Figure 6, Table S6). The main pathways regulated by FpDREB2A according to KEGG enrichment were plant hormone signal transduction, plant–pathogen interaction, transcription factors, protein kinases, glycolysis/gluconeogenesis, phenylalanine metabolism, phenylpropanoid biosynthesis, starch and sucrose metabolism, arginine and proline metabolism and flavonoid biosynthesis, stilbenoid, diarylheptanoid and gingerol biosynthesis (Figure 8).

ABA moderate concentration provoked root elongation (Hodge *et al*., 2009) and enhanced drought resistance (Israelsson *et al*., 2006), but increased ABA concentration inhibited GA accumulation in response to water‐deficit stress (Sun, 2010). Four DEGs, PYR/PYL, PP2C, SnRK2 and ABF (Figure 7) act as a minimal set of DEGs for complete ABA signalling pathway (Fujii *et al*., 2009). Stochasticity in gene expression was observed in the case of PYR/PYL which acts as ABA receptors (Takeuchi *et al*., 2014) showed down‐regulation by RT‐qPCR while, up‐regulation by KEGG annotation. The up–down regulation provide the flexibility needed by cells to adapt to fluctuating environments or respond to sudden stresses, and a mechanism by which population heterogeneity can be established during cellular differentiation and development (Kaern *et al*., 2005). ABA was up‐regulated while GA level decreased in transgenic plant roots (Figure 4d,f), as up‐regulation of DELLA acts as repressor and down‐regulation of GID1 acts as receptor in GA signalling pathways (Sun, 2010, 2011; Ubeda‐Tomas *et al*., 2008). The diverse changes in ABA and GA levels of transgenic black locust plant roots coincided response of plant to drought stress (Krugman, 2013).

DEGs in auxin pathway revealed the up‐regulation of *SAUR* and down‐regulation of *GH3*. GH3 protein catalyses IAA–amino acid conjugates; down‐regulation of *GH3* reduced its negative effect on primary and lateral root formation and growth; and similar results are discussed earlier in rice and *Arabidopsis thaliana* (Fu *et al*., 2011; Khan and Stone, 2007). The up‐regulation of *SAUR* has positive effect on IAA synthesis, and its transport to root tip. High IAA level (Figure 4e) involved reasonably in root elongation, lateral root development and gravity responses (Strohm *et al*., 2012; Xu *et al*., 2013).

Cytokinin produced mainly in roots and negatively regulated root growth and branching (Bhargava and Sawant, 2013). In the present works, up‐regulation *A*‐*ARR* which acted as negative regulator in cytokinin signal transduction (Hirose *et al*., 2007; Hwang and Sakakibara, 2006; Kiba *et al*., 2003), as a result ZR contents were found lower in transgenic plant roots than that of WT plants. The cytokinin degradation or reduced concentration provokes root growth and branching in response to drought stress (Werner *et al*., 2010). The transcription factor EIN3 in ethylene signalling pathway was negatively regulated by the transcriptional regulator EBF1/2 (Figure 8). The down‐regulation of EBF1/2 plays an important role in ethylene‐related gene expression and provokes root development (Zhu *et al*., 2011). The increase in ABA and IAA contents and decrease in GA and ZR levels combined with the changes of DEGs in the roots, brought about by the insertion of the *FpDREB2A* gene involved in the regulation of root architecture, with horizontal and vertical stretches, in particular. Hormone signalling plays diverse and critical roles during plant development. In particular, hormone interactions regulate meristem function, thereby controlling the formation of all organs in the plant.

Analysis of the root transcriptome of *FpDREB2A* transgenic *R. pseudoacacia ‘*Idaho’ showed change in gene expression pathways, the modified DEGs in pathways include plant hormone signalling, cell wall construction, glycolysis, protein and energy metabolism involved in the root architecture. These modified pathways in plant hormone signalling are assumed to be the main cause of horizontal and vertical root development, in particular. The *FpDREB2A* gene confirmed its potential to enhance stress resistance to drought and improve vertical root development in black locust plants.

## Experimental procedures

Three drought‐resistant *DREB* genes *FpDREB2A, MtDREB1A* and *MtDREB1C* were used in this study. *DREB* gene *FpDREB2A* was isolated from *Fraxinus pennsylvanica* Marshvar. *subintegerrima* (Vahl.) Fern. *MtDREB1A* and *MtDREB1C* from *Medicago truncatula* Gaertn, the DNA sequences have been submitted to NCBI GenBank with accession numbers AY536056, DQ778006 and DQ267620, respectively (Zeng *et al*., 2004).

The yeast strain DRE (carrying reporter genes *HIS3* and *lacZ* driven by the wild type *rd29A* promoter containing the DRE sequence TACCGACAT) and the mDRE (with mutated DRE sequence TATTTTCAT) were provided by Professor Shouyi Chen from the Institute of Genetics and Developmental Biology, Chinese Academy of Science.

### Plant material and growth conditions

*Robinia pseudoacacia* L. ‘Idaho’ (WT) as a mother plants for propagation was provided by Deyou Sun from Institute of Horticultural Specialty, Jian city, Liaoning province of China. The plants were propagated by root cuttings. Selected lines were grown and maintained in field. Tender stems of root‐suckered *R. pseudoacacia* ‘Idaho’ were taken as explants to establish tissue culture and *in vitro* regeneration (Xie *et al*., 2006). Leaves of *in vitro* plantlets were used as receptor materials for genetic transformation.

### DNA binding and transcriptional activation activity analysis of the three groups of genes

Plasmid pAD‐*DREBs* (pAD‐*FpDREB2A*, pAD‐*MtDREB1A* and pAD‐*MtDREB1C*) and pAD (as control) were used to test the binding activities of three DREBs. The four plasmids were introduced into the yeast DRE and mDRE strains using the PEG/LiAc method (Gietz *et al*., 1995) to assess DREB binding to the DRE/mDRE sequence. Furthermore, pBD (control), pBD‐*GAL4‐BD23* and pBD‐*DREBs* (pBD‐*FpDREB2A*, pBD‐*MtDREB1A* and pBD‐*MtDREB1C*) were used to test the transcriptional activation activities of three DREBs and introduced into yeast strain YRG‐2.

### Yeast one‐hybrid and β‐galactosidase activity

DRE‐binding properties and transcriptional activation activities of the three *DREB* genes were identified in eukaryote using yeast one‐hybrid method (Wang and Reed, 1993). Transformed yeast strains were inoculated on selective YPAD medium (without His but containing 10 mm 3‐AT) and nonselective YPAD medium at 30 °C for 2–4 days until the colonies grown about 1–3 mm in diameter. The selective YPAD medium contained 6.7 g/L yeast nitrogen base without amino acids, 100 mL 10 × Dropout Solution, 20 g/L agar, and 20 g/L glucose, and medium was regulated at pH 5.8 with 1 M HCl‐NaOH, and the nonselective YPAD medium contained 20 g/L difcopeptone, 10 g/L yeast extracts, 20 g/L dextrose, 100 mg/L adenine sulphate and 20 g/L agar at pH 6.0.

Colony‐lift filter assay was employed to test the binding and transcriptional activation activities of the *DREBs* and β‐Gal assay (*o*‐nitrophenyl β‐d‐galactoside, ONPG) was used for quantitative detection of the transcriptional activation activities (Breeden and Nasmyth, 1985, 1987). The enzyme activity of galactosidase was calculated as follows:

$$\beta -D-\text{galactosidaseactivity}\;\left(U\right)=\frac{1000\times {\text{OD}}_{420}}{V\times t\times {\text{OD}}_{600}}$$

where OD_420_ is the absorbance after reaction, *V* is the reaction system volume, *t* is the reaction time (min) and OD_600_ is the yeast strain culture medium's absorbance.

### *Agrobacterium tumefaciens*‐mediated FpDREB2A gene transformation

*Agrobacterium tumefaciens* strain GV3101 was provided by Professor Mengzhu Lu from the Research Institute of Forestry, Chinese Academy of Forestry and plasmid pBin438‐*P*_*35S*‐*35S*_‐*GUS* was provided by Professor Shouyi Chen from the Institute of Genetics and Developmental Biology, Chinese Academy of Science. The *GUS* gene was substituted by *FpDREB2A* in plasmid pBin438 (Figure 2a) and the plasmid pBin438‐*P*_*35S*‐*35S*_‐*FpDREB2A* was transformed into GV3101 via freeze‐thaw method (Holsters *et al*., 1978).

GV3101/pBin438‐*P*_*35S*‐*35S*_‐*FpDREB2A* and blank pBin438 vector (control) were transformed into *R. pseudoacacia* ‘Idaho’ (Zeng and Wang, 2006). The G418‐resistant shoots were tested by PCR (Table S1). PCR‐positive transgenic plants were then assessed by Southern blotting. The genomic DNA was extracted from *R. pseudoacacia* ‘Idaho’ leaves using CTAB method and digested with restriction enzyme *Sma*I. Probes for the Southern blotting were labelled with digoxin according to the random primed labelling technique followed by manufacturer's instruction (DIG‐High Prime DNA Labeling and Detection Starter Kit I1745832, Roche Company, Germany).

### Drought resistance test and physiological analysis

Root cuttings with 10 cm in length and 0.8 cm in diameter were taken from WT and transgenic plants and placed in sand medium containers (5 × 8 cm) to propagate young plants. After 3 month, the root suckers with the same height (about 20 cm) and diameter (about 0.5 cm) were transplanted into soil within a box (60 × 40 × 20 cm). The plants were watered for 7 days after transplanted into the soil for revival, then were subjected several times to drought stress by soil drying and re‐watering treatments, that is withholding water till permanent wilting of top meristematic tissues followed by re‐watering in greenhouse (Wang *et al*., 1999, 2006). Leaf samples from 4 different stress stages were collected, and subsequently, physiological characteristics such as chlorophyll contents (Gao, 2006), proline (Li, 2006), soluble sugars (Sun, 2006) and MDA (Feng and Hu, 2006) were assayed in transgenic and WT plants under both normal and drought‐stressed conditions.

### Detection of transgenes and endogenous hormones in the transgenic plants

*FpDREB2A* transgenic *R. pseudoacacia* ‘Idaho’ lines grew in a field followed the guidelines and procedures approved by government accordance “the Regulation on Administration of Agricultural Genetically Modified Organisms Safety” issued in 2001 by the State Council of PR China. The average annual precipitation (494 mm), annual temperature (8.5 °C), the absolute lowest temperature (−33 °C), the absolute highest temperature (39 °C) and frost‐free season (152–175 days) were prevailed in the field trial area during the plant growth.

Samples of roots, stems and leaves from 5‐year‐old transgenic *R. pseudoacacia* ‘Idaho’ were collected and utilized for DNA and RNA extraction and assessed by PCR and RT‐PCR, respectively. Genome DNA was extracted with CTAB method, as a template used for the PCR reaction (Table S1). Total RNA was extracted (Chang *et al*., 2012), treated with RNase‐free DNase (Promega Company, Fitchburg, WI), reverse‐transcribed into cDNA using a cDNA Synthesis Kit (Promega Company) and used as a template for RT‐PCR to test relative transcript levels of *FpDREB2A*. β*‐Actin* was used as an internal control, the primers of β*‐Actin* and *FpDREB2A* were shown in Table S1.

Random root samples of *FpDREB2A* transgenic *R. pseudoacacia* ‘Idaho’ were taken to determine the plant hormones, GA, IAA, ZR and ABA contents with enzyme‐linked‐immunosorbent assay method (ELISA) (Eberle *et al*., 1986; Wu *et al*., 1988). The concentrations of plant hormones were based on fresh weight (Li *et al*., 2013b), each sample was run in triplicate.

### Transcriptome sequence analysis of transgenic and WT *R. pseudoacacia* ‘Idaho’

Total RNA was extracted from the phloem of vertical roots of the 5‐year‐old transgenic *R. pseudoacacia* ‘Idaho’ and horizontal roots of the WT. After the samples enriched by Oligo (dT), two cDNA libraries constructed from mRNA were sequenced by Illumina HiSeq^™^ 2000. The RNA samples were sequenced by Beijing Biomaker Technologies Co, LTD.

The SOAP‐*de novo* software (Li *et al*., 2009) and Trinity software (Grabherr *et al*., 2011) were used for *de novo* assembly of the clean reads to generate contigs and transcripts, respectively. The transcripts were clustered to obtain unigene which was the longest among the similar transcripts. All unigenes were aligned to public databases (including NR, NT, Swiss‐Prot, TrEMBL, GO, COG and KEGG) by BLAST to get gene functional annotations.

The expression level of each unigene sequence was measured through FPKM values (Trapnell *et al*., 2010). Differential expressions of the similar unigenes were measured by calculating the ratio of FPKM and the DEGs across the two samples were identified by applying screening thresholds of twofold changes with IDEG 6 software (Romualdi *et al*., 2003). GO, COG terms and KEGG pathways enrichment analysis was used for functional categorization of DEGs.

### RT‐qPCR analysis of DEGs

Total RNA was isolated from transgenic and WT and treated with RNase‐free DNase (Chang *et al*., 2012). The RNA samples were reverse‐transcribed with a cDNA Synthesis Kit (CWBIO Inc., Beijing, China). cDNA products were used for SYBR Green‐based RT‐qPCR analysis, each sample was run in triplicate. The RT‐qPCR running conditions were as follows: 95 °C for 10 min, followed by 40 cycles of 95 °C for 15 s, 52 °C for 20 s, and 72 °C for 30 s, with a final step of 72 °C for 10 min. Using the roots of WT as a standard (control), the expression levels of DEGs in the transgenic plant roots were calculated using the 2^−∆∆Ct^ method (Livak and Schmittgen, 2001).

### Data analysis

Data were subjected to analysis of variance (ANOVA), and differences between means were evaluated with Student's *t*‐test. The differences were considered statistically significant when *P *<* *0.01.

## Supporting information


**Figure S1** WT and transgenic *R. pseudoacacia* ‘Idaho’ were planted in a field after 5 years.

**Figure S2** Length distribution of unigene in WT (T_1_) and transgenic (T_2_) *R. pseudoacacia* ‘Idaho’ cDNA libraries.

**Figure S3** Functional classifications of GO terms of all *R. pseudoacacia* ‘Idaho’ unigenes.

**Figure S4** COG classification of all *R. pseudoacacia* ‘Idaho’ unigenes.

**Figure S5** KEGG annotation of all *R. pseudoacacia* ‘Idaho’ unigenes.

**Figure S6** Scatter plot showing gene expression quantity in WT and transgenic cDNA libraries.

**Table S1** Primers for PCR, RT‐PCR and RT‐qPCR

**Table S2** Summary of the ORFs forecast from total unigenes

**Table S3** Occurrence of SSRs in *R. pseudoacacia* ‘Idaho’ transcriptome

**Table S4** Top 15 up‐regulated DEGs respond to stress in root of transgenic *R. pseudoacacia* ‘Idaho’ compared with WT by GO database

**Table S5** Regulated DEGs involved in groups of ‘transcription’, ‘signal transduction mechanisms’ and ‘replication, recombination and repair’ in root of transgenic *R*. *pseudoacacia* ‘Idaho’ by COG database

**Table S6** All the enriched pathways in KEGG database in roots of WT and transgenic *R*. *pseudoacacia* ‘Idaho’

**Table S7** DEGs involved in plant hormone signal transduction in root of WT and transgenic *R*. *pseudoacacia* ‘Idaho’

**Table S8** Important DEGs response to overexpression of *FpDREB2A* in transgenic plant root
