# Supplementary figures and images for: Improvement and transcriptome analysis of root architecture by overexpression of Fraxinus pennsylvanica DREB2A transcription factor in Robinia pseudoacacia L. ‘Idaho’
Source: Plant Biotechnol J. 2016 Jan 25;14(6):1456–69. doi: 10.1111/pbi.12509 (PMC5066641; doi:10.1111/pbi.12509)

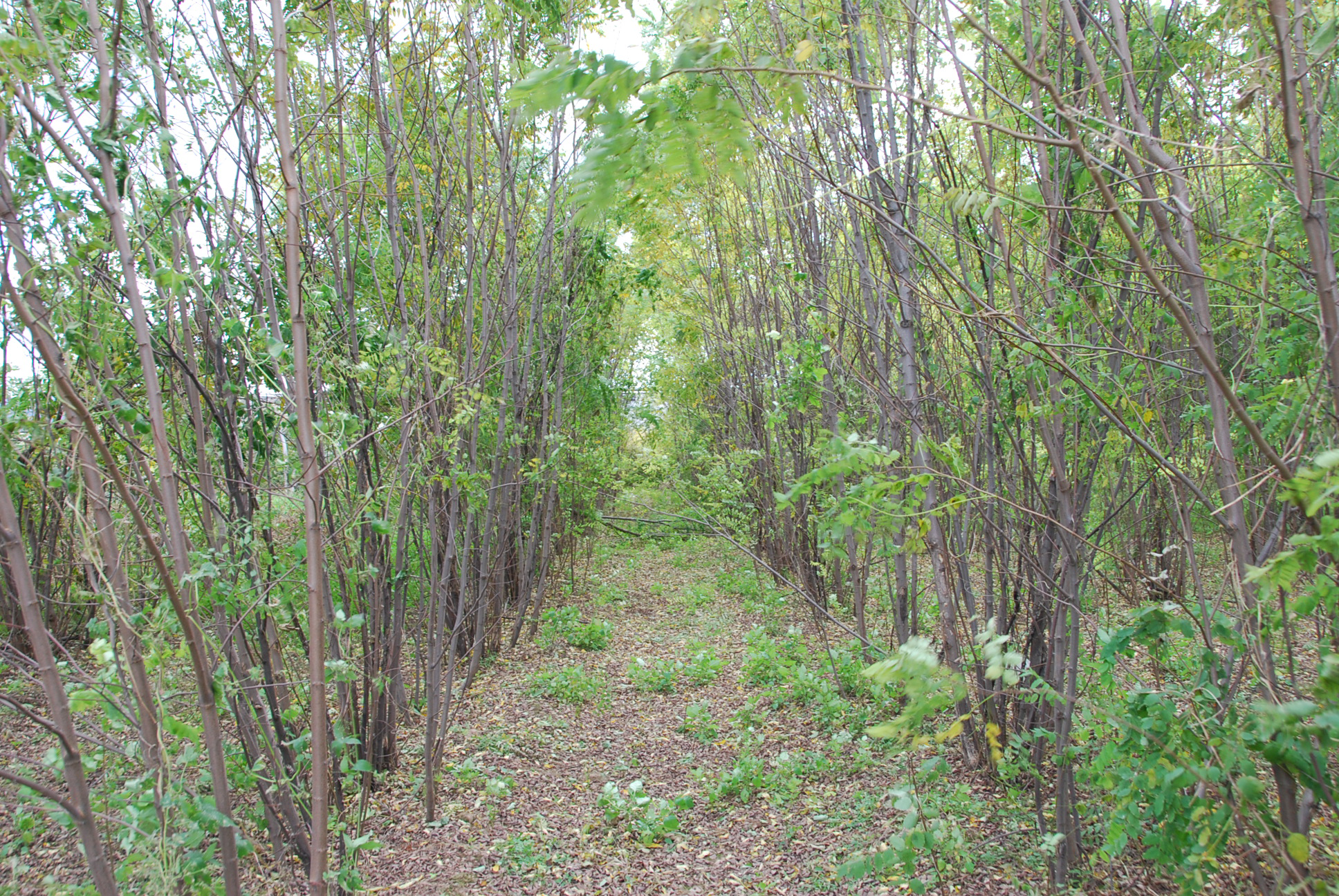

Supplement: Supplementary file 1 — Figure S1 WT and transgenic R. pseudoacacia ‘Idaho’ were planted in a field after 5 years. [file PBI-14-1456-s001.tif]

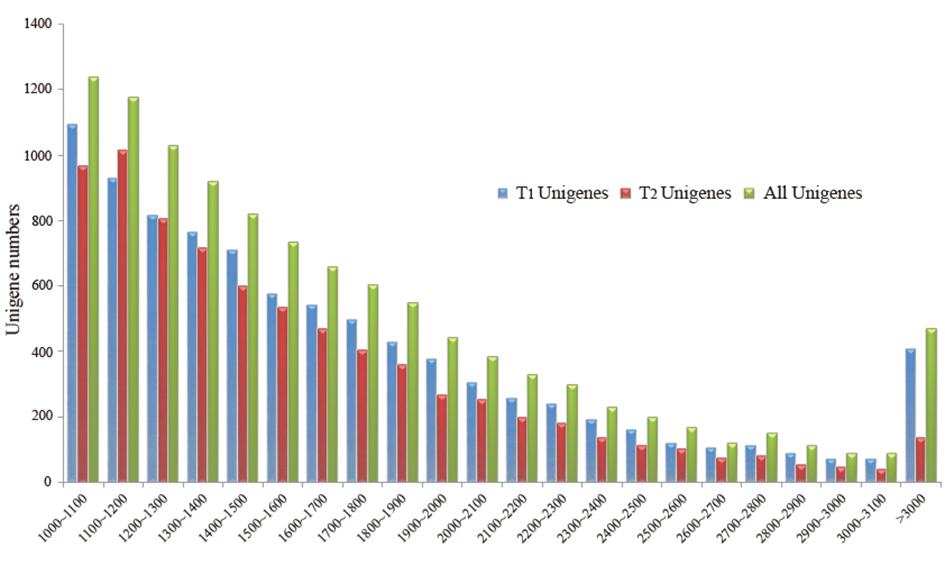

Supplement: Supplementary file 2 — Figure S2 Length distribution of unigene in WT (T1) and transgenic (T2) R. pseudoacacia ‘Idaho’ cDNA libraries. [file PBI-14-1456-s012.tif]

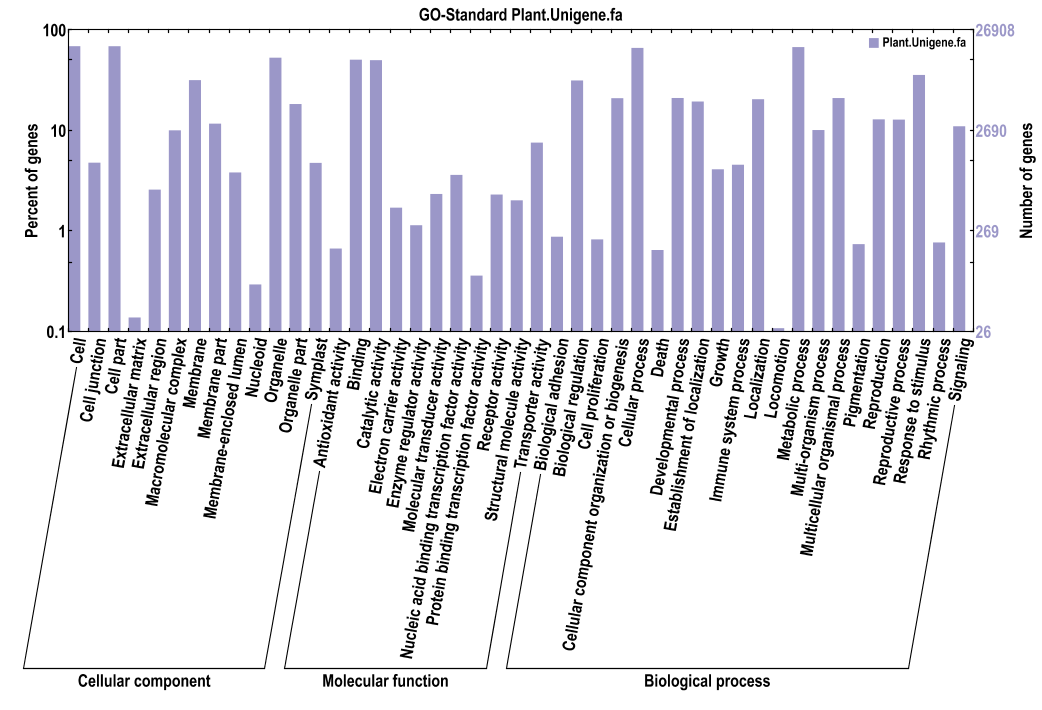

Supplement: Supplementary file 3 — Figure S3 Functional classifications of GO terms of all R. pseudoacacia ‘Idaho’ unigenes. [file PBI-14-1456-s011.tif]

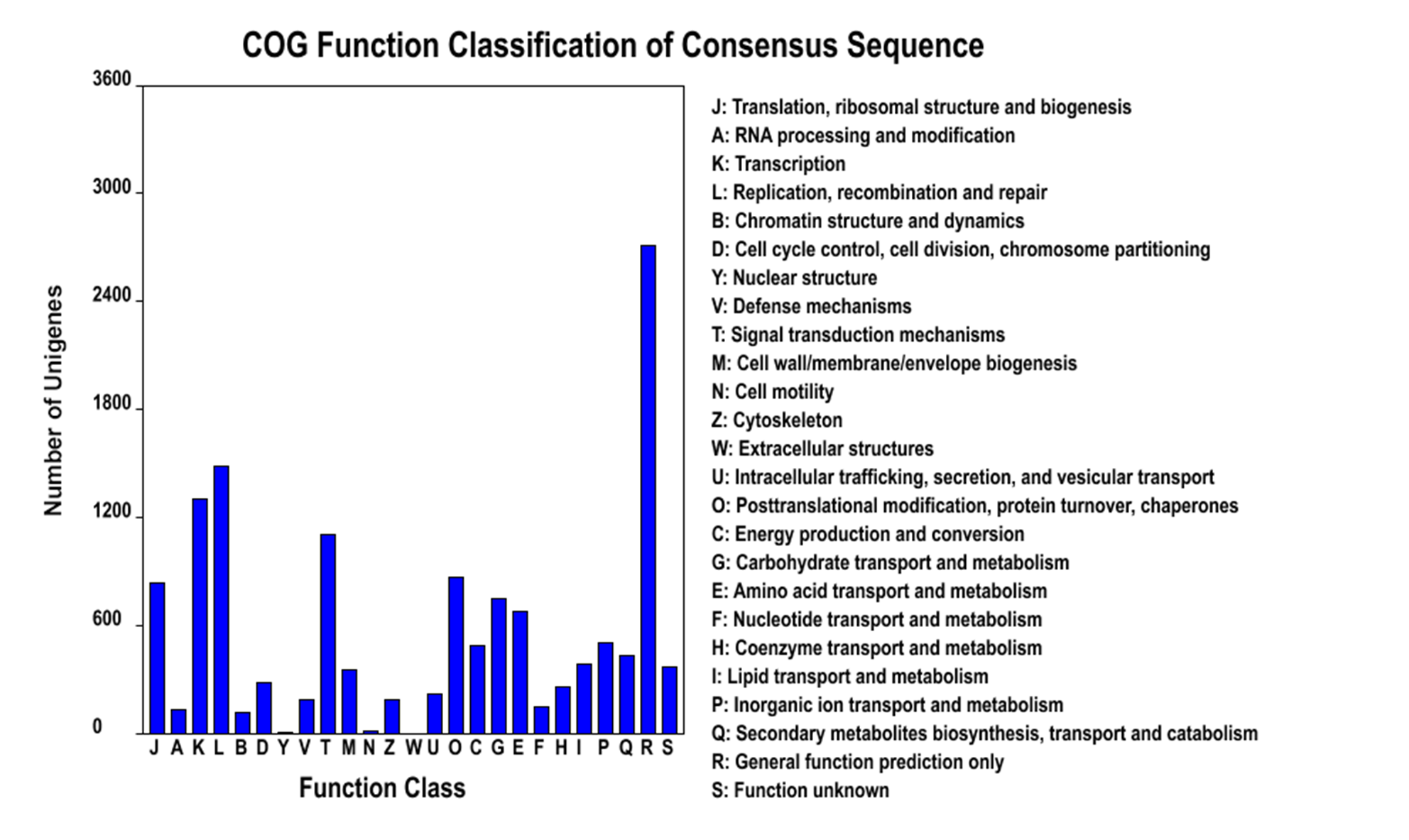

Supplement: Supplementary file 4 — Figure S4 COG classification of all R. pseudoacacia ‘Idaho’ unigenes. [file PBI-14-1456-s013.tif]

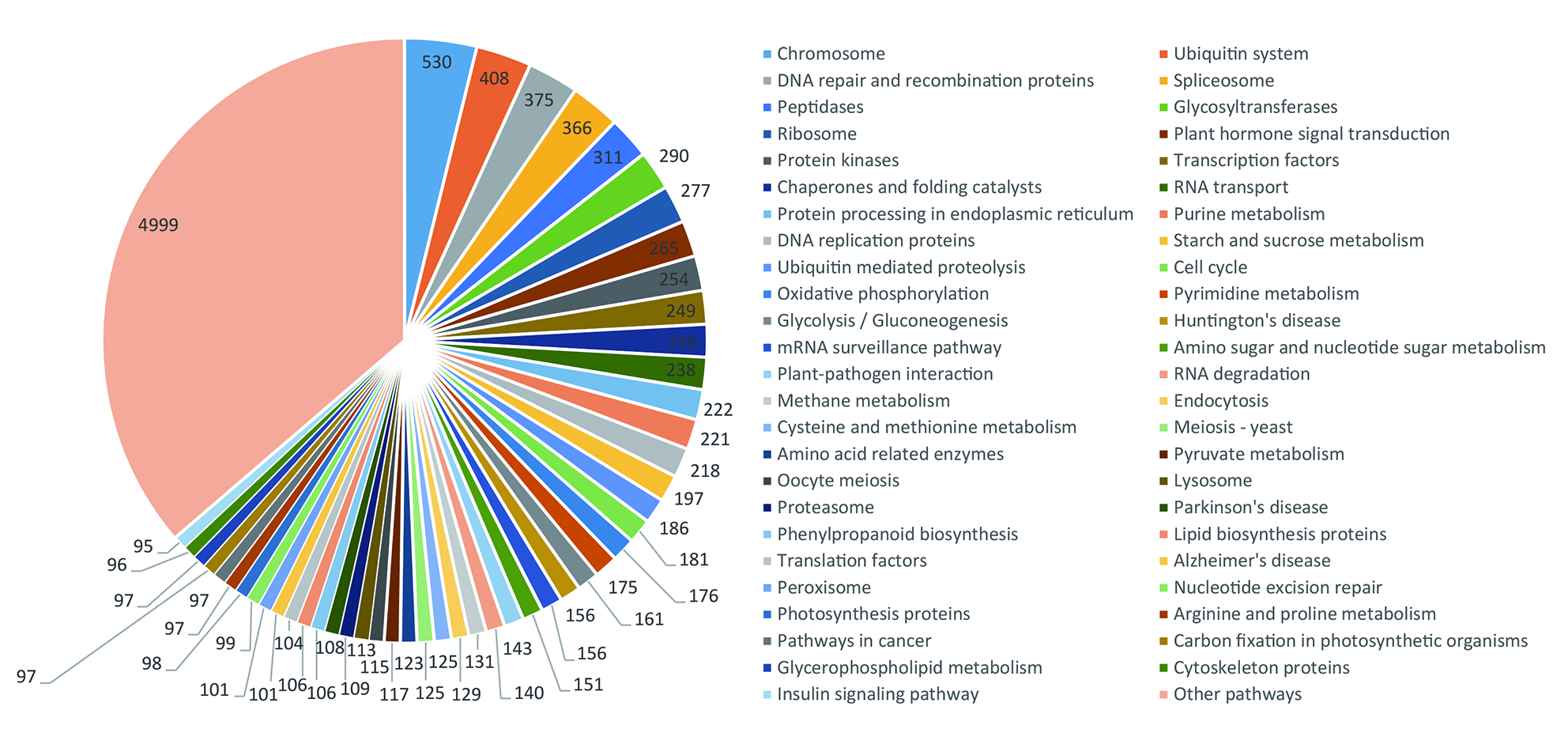

Supplement: Supplementary file 5 — Figure S5 KEGG annotation of all R. pseudoacacia ‘Idaho’ unigenes. [file PBI-14-1456-s014.tif]

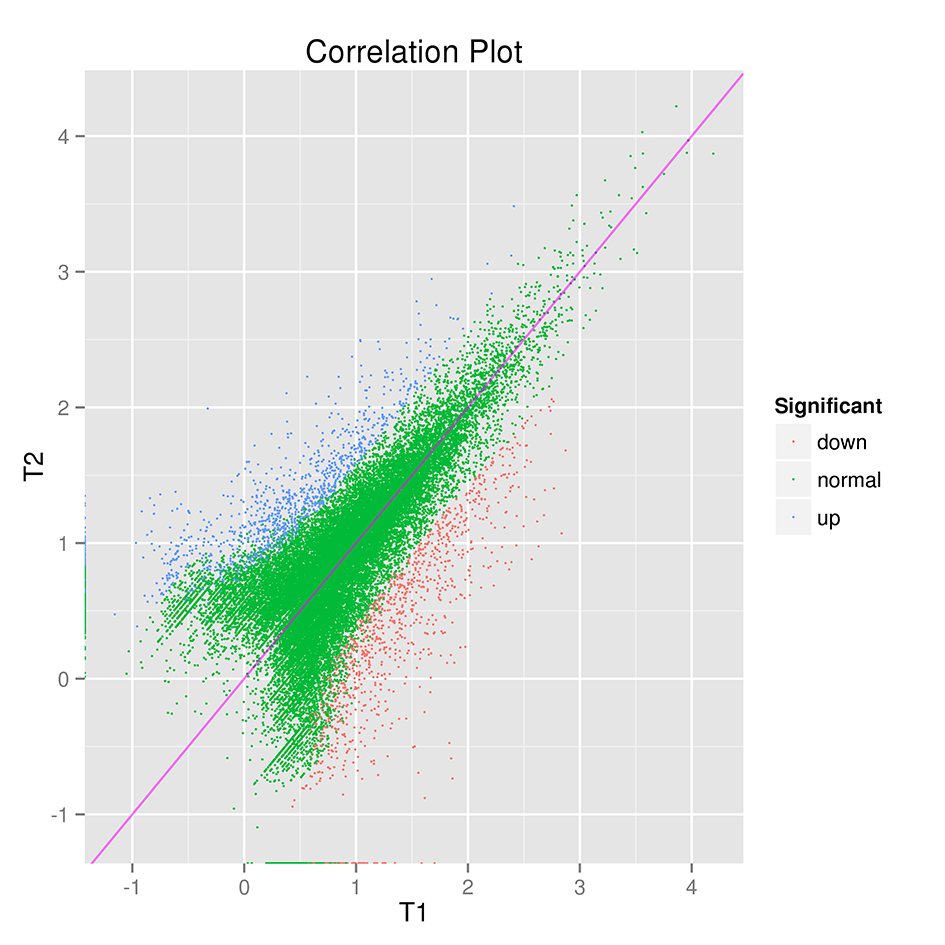

Supplement: Supplementary file 6 — Figure S6 Scatter plot showing gene expression quantity in WT and transgenic cDNA libraries. [file PBI-14-1456-s002.tif]
