# Supplementary material for: Improvement and transcriptome analysis of root architecture by overexpression of Fraxinus pennsylvanica DREB2A transcription factor in Robinia pseudoacacia L. ‘Idaho’
Source: Plant Biotechnol J. 2016 Jan 25;14(6):1456–69. doi: 10.1111/pbi.12509 (PMC5066641; doi:10.1111/pbi.12509)
Supplement: Supplementary file 7 — Table S1 Primers for PCR, RT‐PCR and RT‐qPCR [file PBI-14-1456-s003.docx]

Table S1 Primers for PCR, RT-PCR and RT-qPCR

|  | Primers |
| --- | --- |
| PCR and RT-PCR for *FpDREB2A* | S: 5’-CCGGTGGAGTGGAGCCGATGT-3’  A: 5’-TCCCTCGAGCTGAAACGGAGGT-3’ |
| RT-PCR and RT-qPCR for *β-Actin* | S: 5’-GCCATCTTTGATTGGAATGG-3’  A: 5’-GGTGCCACAACCTTGATCTT-3’ |
| RT-qPCR for GH3 | S: 5’-TTGCTACCCTTTCCAGTCCC-3’ |
|  | A: 5’-AATCCCACCCCAGTTCTTACC-3’ |
| RT-qPCR for SAUR | S: 5’-CCTTTTTCCACACTCCACTAC-3’ |
|  | A: 5’-GCACGATGAACCTCCTCTTA-3’ |
| RT-qPCR for A-ARR | S: 5’-TGAAAGGTGAAGTGAAGGAA-3’ |
|  | A: 5’-GGTGTGGCAAAAATGGAGAC-3’ |
| RT-qPCR for GID1 | S: 5’-AGGACAGGATAGAGACCAT-3’ |
|  | A: 5’-GGCAAGAAGTAGAAACCAA-3’ |
| RT-qPCR for DELLA | S: 5’-TTGAGATGGTTATGGGTAGTGC-3’ |
|  | A: 5’-GGTCGTTGATTTGAGGGTTT-3’ |
| RT-qPCR for PYL | S: 5’-TGGCATTGGAAGTGTTAGAG-3’ |
|  | A: 5’-CAAGTTTCATCCCTGGTGTT-3’ |
| RT-qPCR for PP2C | S: 5’-GTGCTTGGGGTGTTGGCTAT-3’ |
|  | A: 5’-CATCCTCGTCATTCCGTTCC-3’ |
| RT-qPCR for SnRK2 | S: 5’-TAATGTCCCCAATGCTAATG-3’ |
|  | A: 5’-GGAGGGCACACAAAATCAC-3’ |
| RT-qPCR for ABF | S: 5’-GGGAGGGATGATTGGAGT-3’ |
|  | A: 5’-CTGGTGACAGTGAAGAGGTAT-3’ |
