# Supplementary material for: Improvement and transcriptome analysis of root architecture by overexpression of Fraxinus pennsylvanica DREB2A transcription factor in Robinia pseudoacacia L. ‘Idaho’
Source: Plant Biotechnol J. 2016 Jan 25;14(6):1456–69. doi: 10.1111/pbi.12509 (PMC5066641; doi:10.1111/pbi.12509)
Supplement: Supplementary file 8 — Table S2 Summary of the ORFs forecast from total unigenes [file PBI-14-1456-s004.docx]

Table S2 Summary of the ORFs forecast from total unigenes

| Length distribution of ORF (aa) | Number |
| --- | --- |
| ≥1000 | 5,658 (10.11%) |
| 500～999 | 7,671 (13.71%) |
| 200～499 | 15,584 (27.86%) |
| ＜200 | 27,030 (48.32%) |
