# Supplementary material for: Improvement and transcriptome analysis of root architecture by overexpression of Fraxinus pennsylvanica DREB2A transcription factor in Robinia pseudoacacia L. ‘Idaho’
Source: Plant Biotechnol J. 2016 Jan 25;14(6):1456–69. doi: 10.1111/pbi.12509 (PMC5066641; doi:10.1111/pbi.12509)
Supplement: Supplementary file 9 — Table S3 Occurrence of SSRs in R. pseudoacacia ‘Idaho’ transcriptome [file PBI-14-1456-s005.docx]

Table S3 Occurrence of SSRs in *R. pseudoacacia* ‘Idaho’ transcriptome

| Searching item | Numbers |
| --- | --- |
| Total number of sequences examined | 10,786 |
| Total number of identified SSRs | 4,361 |
| Number of sequences containing SSR | 3,314 |
| Number of sequences containing more than 1 SSR | 823 |
| Number of SSRs present in compound formation | 264 |
| Mono-nucleotide | 2,241 (51.39%) |
| Di-nucleotide | 992 (22.75%) |
| Tri-nucleotide | 1,072 (24.59%) |
| Tetra-nucleotide | 38 (0.87%) |
| Penta-nucleotide | 10 (0.23%) |
| Hexa-nucleotide | 8 (0.18%) |
